# Retraction Note: Systems engineering of *Escherichia coli* for high-level glutarate production from glucose

**DOI:** 10.1038/s41467-024-53070-2

**Published:** 2024-10-09

**Authors:** Zhilan Zhang, Ruyin Chu, Wanqing Wei, Wei Song, Chao Ye, Xiulai Chen, Jing Wu, Liming Liu, Cong Gao

**Affiliations:** 1 School of Biotechnology and Key Laboratory of Industrial Biotechnology of Ministry of Education, Jiangnan University, Wuxi, 214122 China; 2 School of Life Sciences and Health Engineering, Jiangnan University, Wuxi, 214122 China; 3 School of Food Science and Pharmaceutical Engineering, Nanjing Normal University, Nanjing, 210000 China

**Keywords:** Applied microbiology, Enzyme mechanisms, Metabolic engineering, Metabolic pathways

Retraction to: *Nature Communications* 10.1038/s41467-024-45448-z, published online 03 February 2024

The Editors have retracted this article at the request of the authors. After publication, the authors were informed of concerns regarding the activity of the final enzyme of the newly constructed pathway for glutarate production from glucose. The enzyme (*Kp*ALDH) was derived from *Klebsiella pneumoniae* and initially considered to have the aldehyde dehydrogenase activity. However, based on sequence comparison and computational modeling of structure and reaction mechanism, *Kp*ALDH is closely related to macrolide phosphotransferase rather than aldehyde dehydrogenase. As a result, the authors repeated experiments for the enzymatic activity of *Kp*ALDH and concluded that *Kp*ALDH cannot catalyze the synthesis of glutarate from glutaraldehyde, as had been reported in this paper. In addition, the authors found that the product of their fermentation experiment was not glutarate after optimizing the HPLC detection method. As these were the key findings of this article, the authors consider that the conclusions of this work are unsupported. The authors would like to apologize for any inconvenience caused to the readers of the journal.

